# High Mortality Rate in Oral Glucocorticoid Users: A Population-Based Matched Cohort Study

**DOI:** 10.3389/fendo.2022.918356

**Published:** 2022-07-08

**Authors:** Margret J. Einarsdottir, Per Ekman, Mattias Molin, Penelope Trimpou, Daniel S. Olsson, Gudmundur Johannsson, Oskar Ragnarsson

**Affiliations:** ^1^ Department of Internal Medicine and Clinical Nutrition, Institute of Medicine at Sahlgrenska Academy, University of Gothenburg, Gothenburg, Sweden; ^2^ Department of Endocrinology, Sahlgrenska University Hospital, Gothenburg, Sweden; ^3^ Statistiska Konsultgruppen, Gothenburg, Sweden

**Keywords:** glucocorticoids, mortality, adrenal insufficiency, cohort study, corticosteroids

## Abstract

**Objective:**

The aim of the study was to investigate all-cause and disease-specific mortality in a large population-based cohort of oral glucocorticoid (GC) users.

**Methods:**

This was a retrospective, matched cohort study. Information on dispensed prescriptions was obtained from the Swedish Prescribed Drug Register. The cause of death was obtained from the Swedish Cause-of-Death Registry. Patients receiving prednisolone ≥5 mg/day (or equivalent dose of other GC) for ≥21 days between 2007-2014 were included. For each patient, one control subject matched for age and sex was included. The study period was divided into 3-month periods and patients were divided into groups according to a defined daily dose (DDD) of GC used per day. The groups were: Non-users (0 DDD per day), low-dose users (>0 but <0.5 DDD per day), medium-dose users (0.5-1.5 DDD per day) and high-dose users (>1.5 DDD per day). Hazard ratios (HRs), unadjusted and adjusted for age, sex and comorbidities, were calculated using a time-dependent Cox proportional hazard model.

**Results:**

Cases (n=223 211) had significantly higher all-cause mortality compared to controls (HR adjusted for age, sex and comorbidities 2.08, 95% confidence interval 2.04 to 2.13). After dividing the cases into subgroups, adjusted HR was 1.31 (1.28 to 1.34) in non-users, 3.64 (3.51 to 3.77) in low-dose users, 5.43 (5.27 to 5.60) in medium-dose users and, 5.12 (4.84 to 5.42) in high-dose users. The highest adjusted hazard ratio was observed in high-dose users for deaths from sepsis 6.71 (5.12 to 8.81) and pulmonary embolism 7.83 (5.71 to 10.74).

**Conclusion:**

Oral GC users have an increased mortality rate compared to the background population, even after adjustment for comorbidities. High-dose users have an increased risk of dying from sepsis, and pulmonary embolism compared to controls. Whether the relationship between GC exposure and the excess mortality is causal remains to be elucidated.

## Introduction

Glucocorticoids (GCs) are commonly used worldwide for the treatment of various diseases (1, 2). GC treatment is associated with several adverse effects such as osteoporosis, hypertension, insulin resistance, infections, mood disturbances, cataract formation, and increased risk of cardiovascular disease (3–8). Previous studies have shown that GC use is associated with increased all-cause mortality and cardiovascular mortality in patients with rheumatoid arthritis, where both longer duration of treatment and higher doses predict worse outcome (9–11). Similarly, oral GC use is associated with increased mortality in patients with asthma and chronic inflammatory diseases (12–15). Moreover chronic GC users have a 1.4-fold higher 5-year all-cause mortality (16).

Most previous studies on mortality in GC users are limited to patients with specific underlying disease and none has investigated pulmonary embolism as a specific cause of death.

The aim of this study was to investigate all-cause and disease-specific mortality in a large population-based cohort of oral GC users. Our main hypothesis was that mortality in oral GC users is higher than in the background population.

## Methods

This was a retrospective, matched cohort study based on data from five Swedish healthcare registries. Data on GC prescriptions were collected from the Prescribed Drug Register by using the Anatomical Therapeutic Chemical codes for prednisolone, hydrocortisone, betamethasone, and dexamethasone. The Prescribed Drug Register has information on all prescriptions that are dispensed at Swedish pharmacies since July 2005 (17). We included patients living in Västra Götaland County, Sweden, with dispensed equivalent daily oral GCs doses of prednisolone ≥5 mg, hydrocortisone ≥20mg, betamethasone ≥0.5 mg, or dexamethasone ≥0.5 mg, for more than 21 days, from 1 January 2007 to 31 December 2014. For every prescription the number of Defined daily dose (DDD) is registered. In the registry, DDD is defined according to the World Health Organization (WHO) definition (18). For GC the definition was 1 DDD=10mg Prednisolone=1.5 mg Betamethasone=1.5mg Dexamethasone=30mg hydrocortisone. The patients, defined as cases, were divided into four groups according to GC use: 1. Non-users (0 DDD per day). 2. Low-dose users (more than 0 DDD per day but lower than 0.5 DDD per day). 3. Medium-dose users (0.5-1.5 DDD per day) 4. High-dose users (more than 1.5 DDD per day). A case may at any time point in the study move between those DDD groups based on the previous 3-month period. For example, one case could be classified as a high user in the beginning of the study and at a later time point as a non-user, depending on the GC dose dispensed during the previous 3 months. A non-user is a case that meets the inclusion criteria and has dispensed at least one prescription for GC but has not dispensed GC in the previous 3 months.

For each case, one control subject from Västra Götaland’s population register (Västfolket), matched for age (same year of birth) and sex, was included. Controls with any dispensed systemic GC (i.e., tablets or injections) during 2005-2014 were excluded. In cases, the date of inclusion was defined as the date of the first dispensed GC prescription. Follow-up time was calculated from study inclusion to death or end of study (December 31, 2014). For controls, the same inclusion date was used as for their matched GC user.

By using a personal identification number, cases and controls were cross-linked with the Swedish Cause-of-Death Registry. Information on date of death, the primary cause of death, and contributing causes of death were collected and used for the mortality analysis. All-cause mortality and mortality due to ten pre-specified diseases (i.e., ischemic heart disease, myocardial infarction, heart failure, pulmonary embolism, stroke, stroke unspecified, cerebral infarction, intracerebral hemorrhage, sepsis, and pneumonia) were analyzed. A list of the pre-specified diseases, and the corresponding International Classification of Disease 10th edition (ICD-10) codes, is provided in **Table 1**.

**Table 1:** Cause of death and ICD-10 codes.

Cause of death	ICD-10 code
Ischemic heart disease	I20 to I25
Myocardial infarction	I21
Heart failure	I50
Pulmonary embolism	I26
Cerebral infarction	I63
Intracerebral hemorrhage	I61 and I62
Stroke (total)	I61 to I64
Stroke UNS	I64
Sepsis	A40 and A41
Pneumonia	J12 to J18

ICD, International Classification of Diseases; UNS, unspecified.

The Swedish National Patient Register (NPR) and the Västra Götaland Regional Healthcare Database (VEGA) were used to collect information on the indication for GC treatment, and other comorbidities, by gathering ICD-10 codes during a 24-month period prior to the date of inclusion. The NPR contains all diagnoses for inpatients and hospital-based outpatient care in Sweden (19). VEGA comprises information on diagnoses from primary healthcare and private care in Västra Götaland County. Information on cancer diagnoses was collected from the Swedish Cancer Registry that covers all cancer diagnoses in Sweden (20).

### Statistical Analysis

Categorical variables are presented as number and percent and age as mean, standard deviation, median, and range. Years of follow-up are presented as mean, median, and sum. Mortality rate was calculated as number of deaths per follow-up days and then converted to number of deaths per 1000 observation years. Hazard ratio with 95% confidence interval (CI) for mortality in GC users, relative to controls, was evaluated by using Cox proportional hazard models. Difference in survival was determined by the log-rank test. When evaluating mortality by DDD groups time-dependent Cox proportional hazard models presenting hazard ratios with 95% CI and p-values. Both unadjusted hazard ratio, and hazard ratio adjusted for age, sex and comorbidities (diabetes ICD-10 code E10-E14, deep vein thrombosis I80-I82, pulmonary embolism I26, hypertension (more than 2 dispensed prescription of antihypertensive drug), stroke I64, ischemic heart disease I20-I25, heart failure I50, pneumonia J12-J18, malignant neoplasm C00-C97), diagnosed from two years prior to inclusion, were calculated. For the adjusted models the p-value from the Cox model is presented. All significance tests were two-sided and conducted at a 5% significance level. All analyses were performed using SAS^®^ version 9.4 (Cary, NC).

### Ethical Approval

The study was approved by the Regional Research Ethics Committee in Gothenburg, Sweden (reference number 773-14; approved 9 March 2015) and by the National Board of Health and Welfare, Sweden.

## Results

Of 1 585 335 inhabitants in Västra Götaland, 223 211 cases (55.6% women) and an equal number of matched controls were included in the study (**Table 2**). Mean age was 48.4 years (standard deviation 24.2). Chronic obstructive pulmonary disease and asthma were the most common indications for GC treatment (17.2%), followed by allergy (12.5%) and malignant neoplasms (11.5%).

**Table 2:** Baseline characteristics.

	GC users (n = 223 211)	Controls (n = 223 211)
Age (years)		
Mean (standard deviation)	48.4 (24.2),	48.4 (24.2)
Median (range)	50.8 (0.1 to 107)	50.8 (0.0 to 107)
Gender		
Men	99 172 (44.4%)	99 172 (44.4%)
Women	124 039 (55.6%)	124 039 (55.6%)
Comorbidities prior to inclusion*		
Diabetes mellitus	14 249 (6.4%)	12 449 (5.6%)
Heart failure	6898 (3.1%)	3260 (1.5%)
Ischemic heart disease	11 629 (5.2%)	7978 (3.6%)
Hypertension	56 874 (25.5%)	45 896 (20.6%)
Stroke	3418 (1.5%)	2759 (1.2%)
Deep vein thrombosis	2795 (1.3%)	1486 (0.7%)
Pulmonary embolism	1024 (0.5%)	385 (0.2%)
Sepsis	924 (0.4%)	323 (0.1%)
Malignant neoplasm	15927 (7.1%)	3270 (1.5%)

*Comorbidities during a 24-month period prior to the date of inclusion. GC, glucocorticoid.

### All-Cause Mortality

**Figure 1:**
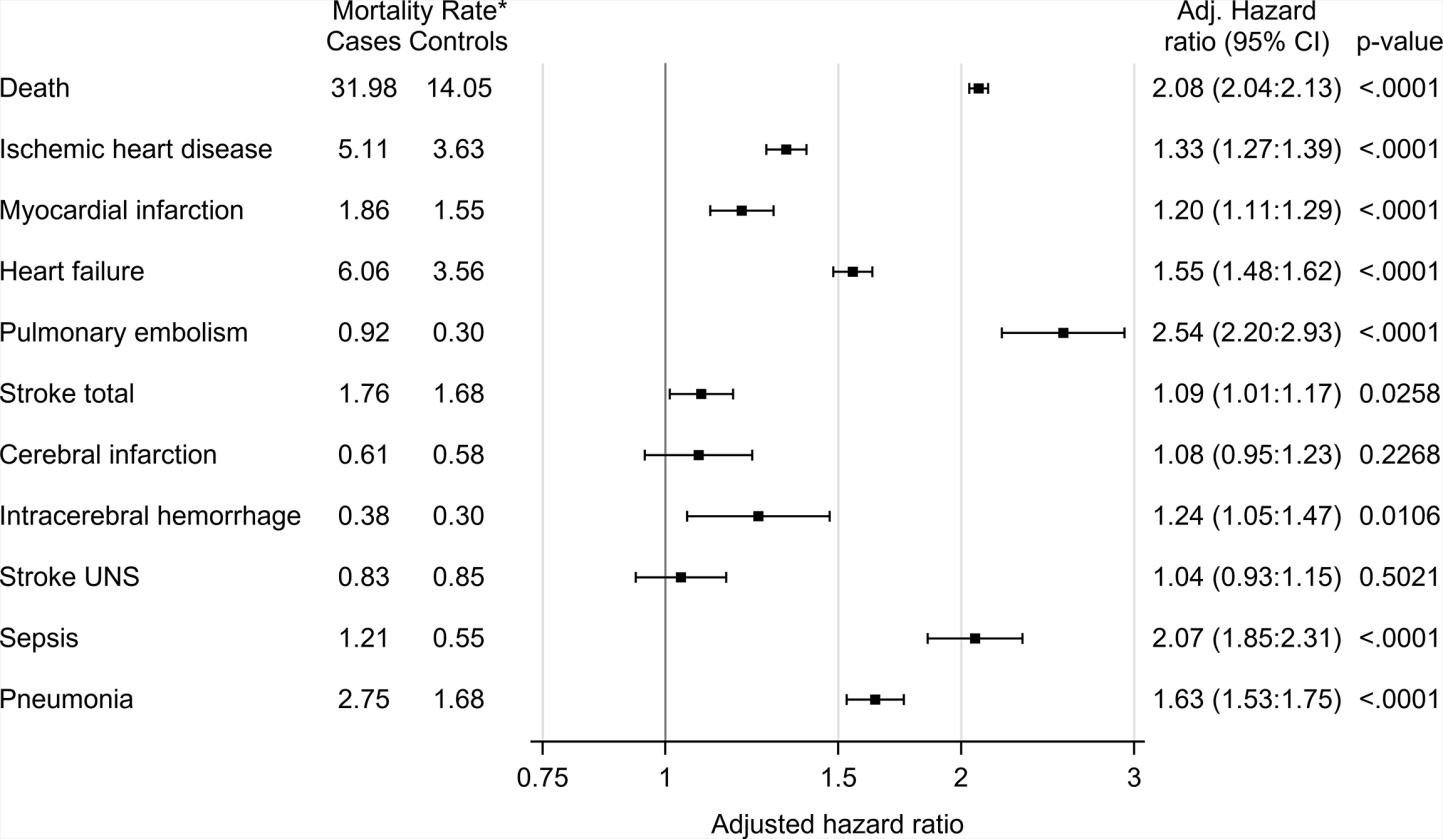
All-cause mortality and disease-specific mortality in glucocorticoid users (cases) compared to age- and sex-matched controls. The hazard ratio was calculated using Cox proportional hazard model. The hazard ratio was adjusted for age, sex and comorbidities (diabetes, deep vein thrombosis, pulmonary embolism, hypertension, stroke, ischemic heart disease, heart failure, pneumonia, malignant neoplasm). *Number of deaths per 1000 patient years.

The mortality rate was 26 550 deaths per 3.6 observation years in cases, compared to 12 384 deaths per 3.9 observation years in controls. The mortality rate per 1000 patient years was 14.05 in controls and 31.98 in cases (**Table 3**). Unadjusted hazard ratio for all-cause mortality in GC users was 2.26 (95% CI 2.21 to 2.30) and after adjustment for age, sex and comorbidities 2.08 (95% CI 2.04 to 2.13) (**Figure 1**). The hazard ratio, adjusted for age, sex and comorbidities, for all-cause mortality in non-users was 1.31 (95% CI 1.28 to 1.34), 3.64 (95% CI 3.51 to 3.77) in low-dose users, 5.43 (95% CI 5.27 to 5.60) in medium-dose users and, 5.12 (95% CI 4.84-5.42) in high-dose users (**Table 4**).

**Table 3:** All-cause mortality and disease-specific mortality in GC users compared to age- and sex-matched controls.

		No of deaths* (%)	Follow-up (years) mean; median; sum	No of deaths per 1000 patient years	Unadjusted		Adjusted for age, sex and comorbidities**	
					Hazard ratio (95% CI)	p-value	Hazard ratio (95% CI)	p-value
Death	Cases	26550 (11.9)	3.72; 3.60; 830294	31.98				
	Controls	12384 (5.5)	3.95; 3.87; 881731	14.05	2.26 (2.21-2.30)	<.0001	2.08 (2.04-2.13)	<.0001
Ischemic heart disease	Cases	4243 (1.9)	3.72; 3.60; 830294	5.11				
	Controls	3205 (1.4)	3.95; 3.87; 881731	3.63	1.40 (1.34-1.47)	<.0001	1.33 (1.27-1.39)	<.0001
Myocardial infarction	Cases	1547 (0.7)	3.72; 3.60; 830294	1.86				
	Controls	1368 (0.6)	3.95; 3.87; 881731	1.55	1.20 (1.11:1.29)	<.0001	1.20 (1.11:1.29)	<.0001
Heart failure	Cases	5030 (2.3)	3.72; 3.60; 830294	6.06				
	Controls	3139 (1.4)	3.95; 3.87; 881731	3.56	1.69 (1.62:1.77)	<.0001	1.55 (1.48:1.62)	<.0001
Pulmonary embolism	Cases	762 (0.3)	3.72; 3.60; 830294	0.92				
	Controls	265 (0.1)	3.95; 3.87; 881731	0.30	3.02 (2.63:3.48)	<.0001	2.54 (2.20:2.93)	<.0001
Stroke total †	Cases	1462 (0.7)	3.72; 3.60; 830294	1.76				
	Controls	1477 (0.7)	3.95; 3.87; 881731	1.68	1.05 (0.97:1.13)	0.2087	1.09 (1.01:1.17)	0.0258
Cerebral infarction	Cases	510 (0.2)	3.72; 3.60; 830294	0.61				
	Controls	509 (0.2)	3.95; 3.87; 881731	0.58	1.06 (0.94:1.20)	0.3459	1.08 (0.95:1.23)	0.2268
Intracerebral hemorrhage	Cases	314 (0.1)	3.72; 3.60; 830294	0.38				
	Controls	268 (0.1)	3.95; 3.87; 881731	0.30	1.24 (1.05:1.46)	0.0098	1.24 (1.05:1.47)	0.0106
Stroke UNS	Cases	687 (0.3)	3.72; 3.60; 830294	0.83				
	Controls	746 (0.3)	3.95; 3.87; 881731	0.85	0.97 (0.88:1.08)	0.6239	1.04 (0.93:1.15)	0.5021
Sepsis	Cases	1006 (0.5)	3.72; 3.60; 830294	1.21				
	Controls	482 (0.2)	3.95; 3.87; 881731	0.55	2.20 (1.98:2.46)	<.0001	2.07 (1.85:2.31)	<.0001
Pneumonia	Cases	2280 (1.0)	3.72; 3.60; 830294	2.75				
	Control	1481 (0.7)	3.95; 3.87; 881731	1.68	1.63 (1.52:1.74)	<.0001	1.63 (1.53:1.75)	<.0001

*The cause of death can be both the primary cause of death and contributing causes of death, so the total number of deaths is therefore higher than the total deaths due to disease-specific deaths.

**Hazard ratio adjusted for comorbidities (diabetes ICD-10 code E10-E14, deep vein thrombosis I80-I82, pulmonary embolism I26, hypertension (more than 2 dispensed prescription of antihypertensive drug), stroke I64, ischemic heart disease I20-I25, heart failure I50, pneumonia J12-J18, malignant neoplasm C00-C97).

†Cerebral infarction, intracerebral hemorrhage, and stroke not specified as hemorrhage or infarction. CI, confidence interval; GC, glucocorticoid; UNS, unspecified; ICD, International Classification of Disease.

**Table 4:** All-cause mortality in oral glucocorticoid (GC) users compared to age- and sex-matched controls.

		No of deaths (%)	Follow-up (years)	No of deaths per 1000 patient years	Unadjusted		Adjusted for age, sex and comorbidities*	
					Hazard ratio (95% CI)	p-value	Hazard ratio (95% CI)	p-value
All-cause mortality	Controls	12384 (5.5)	881731	14.0				
	Non-users (0 DDD per day)	12191 (5.9)	712482	17.1	1.22 (1.19-1.25)	<.0001	1.31 (1.28-1.34)	<.0001
	Low-dose users (lower than 0.5 DDD per day)	5318 (3.0)	70807	75.1	5.30 (5.11-5.49)	<.0001	3.64 (3.51-3.77)	<.0001
	Medium-dose users (0.5-1.5 DDD per day)	7685 (11.4)	39376	195.2	13.10 (12.72-13.50)	<.0001	5.43 (5.27-5.60)	<.0001
	High-dose users (more than 1.5 DDD per day)	1356 (7.7)	8000	169.5	10.81 (10.22-11.44)	<.0001	5.12 (4.84-5.42)	<.0001

*Hazard ratio adjusted for comorbidities (diabetes ICD-10 code E10-E14, deep vein thrombosis I80-I82, pulmonary embolism I26, hypertension (more than 2 dispensed prescription of antihypertensive drug), stroke I64, ischemic heart disease I20-I25, heart failure I50, pneumonia J12-J18, malignant neoplasm C00-C97).

CI, confidence interval; GC, glucocorticoid; ICD, International Classification of Diseases.

### Disease-Specific Mortality

**Figure 2:**
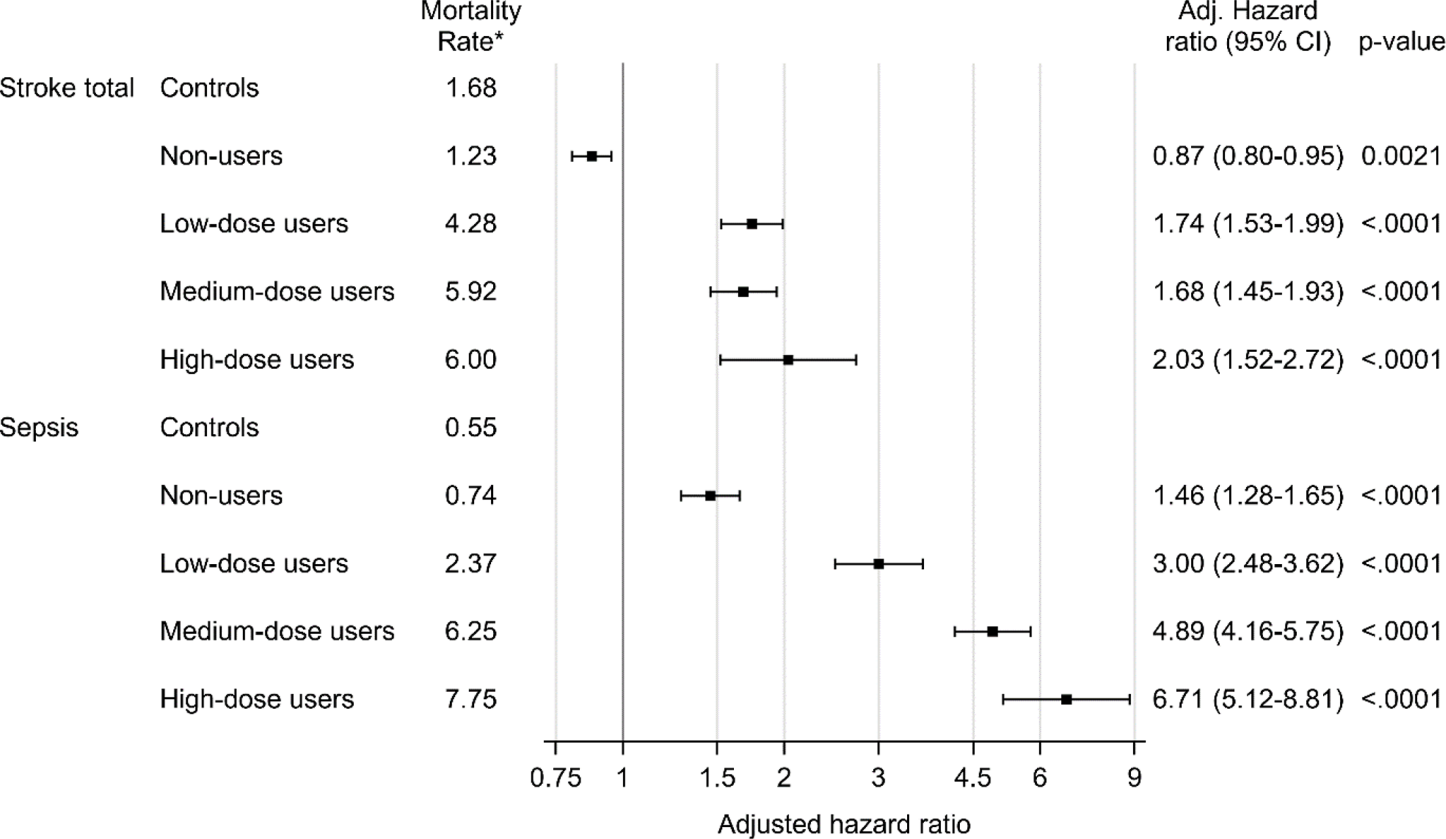
Mortality due to stroke and sepsis in glucocorticoid users compared to age- and sex-matched controls. Mortality analysis was performed for controls and for non-users (0 DDD per day), low-dose users (more than 0 DDD per day but lower than 0.5 DDD per day), medium-dose users (0.5-1.5 DDD per day) and high-dose users (more than 1.5 DDD per day). Time-dependent Cox proportional hazard models was used. The hazard ratio was adjusted for age, sex and comorbidities (diabetes, deep vein thrombosis, pulmonary embolism, hypertension, stroke, ischemic heart disease, heart failure, pneumonia, malignant neoplasm). *Number of deaths per 1000 patient years.

Cox regression was used to analyze mortality due to ten pre-specified diseases (**Table 5**). After adjustment for sex, age and comorbidities, the hazard ratio for death from pulmonary embolism was 1.51 (95% CI, 1.28 to 1.78), 5.16 (95% CI 4.18 to 6.37), 6.77 (95% CI 5.59 to 8.19), and 7.83 (95% CI 5.71 to 10.74) in non-users, low-dose users, medium-dose users and high-dose users, respectively. Mortality from stroke (cerebral infarction, intracerebral hemorrhage or stroke not otherwise specified) was increased in low-dose users, medium-dose users and high-dose users, adjusted hazard ratio 1.74 (95% CI 1.53 to 1.99), 1.68 (95% CI 1.45 to 1.93) and 2.03 (95% CI 1.52 to 2.72), respectively (**Figure 2**). Adjusted hazard ratio for death from sepsis was 1.46 (95% CI 1.28 to 1.65) in non-users, 3.00 (95% CI 2.48 to 3.62) in low users, 4.89 (95% CI 4.16 to 5.75) in medium-dose users and 6.71 (95% CI 5.12 to 8.81) in high-dose users. Increased mortality from pneumonia was found in all GC users´ groups, hazard ratio being 3.34 (95% CI 3.01 to 3.71) in low-dose users, 3.01 (95% CI 2.69 to 3.36) in medium-dose users, and 3.82 (95% CI 3.10 to 4.70) in high-dose users. Adjusted hazard ratio was also increased for death from heart failure, with the highest hazard ratio in low-dose users 2.73 (95% CI 2.54 to 2.93).

**Table 5:** Disease-specific mortality in GC users compared to age- and sex-matched controls.

		No of deaths* (%)	Follow-up (years)	No of deaths per 1000 patient years	Unadjusted		Adjusted for age, sex and comorbidities**	
					Hazard ratio (95% CI)	p-value	Hazard ratio (95% CI)	p-value
Ischemic heart disease	Controls	3205 (1.4)	881731	3.6				
	Non-users (0 DDD per day)	2548 (1.2)	712482	3.6	0.98 (0.93-1.03)	0.35	1.07 (1.01-1.13)	0.014
	Low-dose users (lower than 0.5 DDD per day)	914 (0.5)	70807	12.9	3.81 (3.50-4.14)	<.0001	2.18 (2.01-2.36)	<.0001
	Medium-dose users (0.5-1.5 DDD per day)	678 (1.0)	39376	17.2	4.76 (4.37-5.18)	<.0001	2.09 (1.91-2.27)	<.0001
	High-dose users (more than 1.5 DDD)	103 (0.6)	8000	12.9	3.48 (2.86-4.24)	<.0001	1.88 (1.55-2.30)	<.0001
Myocardial infarction	Controls	1368 (0.6)	881731	1.6				
	Non-users (0 DDD per day)	946 (0.5)	712482	1.3	0.85 (0.78-0.92)	<.0001	0.97 (0.89-1.05)	0.45
	Low-dose users (lower than 0.5 DDD per day)	333 (0.2)	70807	4.7	3.32 (2.90-3.81)	<.0001	2.09 (1.84-2.38)	<.0001
	Medium-dose users (0.5-1.5 DDD per day)	224 (0.3)	39376	5.7	3.72 (3.22-4.30)	<.0001	1.73 (1.50-2.00)	<.0001
	High-dose users (more than 1.5 DDD per day)	44 (0.3)	8000	5.5	3.53 (2.61-4.78)	<.0001	1.97 (1.46-2.67)	<.0001
Heart failure	Controls	3139 (1.4)	881731	3.6				
	Non-users (0 DDD per day)	2931 (1.4)	712482	4.1	1.13 (1.08-1.19)	<.0001	1.22 (1.15-1.28)	<.0001
	Low-dose users (lower than 0.5 DDD per day)	1211 (0.7)	70807	17.1	5.71 (5.30-6.16)	<.0001	2.73 (2.54-2.93)	<.0001
	Medium-dose users (0.5-1.5 DDD per day)	767 (1.1)	39376	19.5	5.73 (5.29-6.21)	<.0001	2.40 (2.21-2.61)	<.0001
	High-dose users (more than 1.5 DDD per day)	121 (0.7)	8000	15.1	4.35 (3.62-5.22)	<.0001	2.43 (2.02-2.92)	<.0001
Pulmonary embolism	Controls	265 (0.1)	881731	0.3				
	Non-users (0 DDD per day)	332 (0.2)	712482	0.5	1.54 (1.31-1.81)	<.0001	1.51 (1.28-1.78)	<.0001
	Low-dose users (lower than 0.5 DDD per day)	163 (0.1)	70807	2.3	8.02 (6.46-9.96)	<.0001	5.16 (4.18-6.37)	<.0001
	Medium-dose users (0.5-1.5 DDD per day)	219 (0.3)	39376	5.6	17.62 (14.65-21.18)	<.0001	6.77 (5.59-8.19)	<.0001
	High-dose users (more than 1.5 DDD per day)	48 (0.3)	8000	6.0	17.78 (13.01-24.28)	<.0001	7.83 (5.71-10.74)	<.0001
Stroke total †	Controls	1477 (0.7)	881731	1.7				
	Non-users (0 DDD per day)	878 (0.4)	712482	1.2	0.73 (0.67-0.79)	<.0001	0.87 (0.80-0.95)	0.0021
	Low-dose users (lower than 0.5 DDD per day)	303 (0.2)	70807	4.3	2.76 (2.40-3.17)	<.0001	1.74 (1.53-1.99)	<.0001
	Medium-dose users (0.5-1.5 DDD per day)	233 (0.3)	39376	5.9	3.59 (3.12-4.14)	<.0001	1.68 (1.45-1.93)	<.0001
	High-dose users (more than 1.5 DDD per day)	48 (0.3)	8000	6.0	3.58 (2.68-4.79)	<.0001	2.03 (1.52-2.72)	<.0001
Cerebral infarction	Controls	509 (0.2)	881731	0.6				
	Non-users (0 DDD per day)	321 (0.2)	712482	0.5	0.78 (0.67-0.89)	0.0004	0.91 (0.79-1.05)	0.21
	Low-dose users (lower than 0.5 DDD per day)	107 (0.1)	70807	1.5	2.73 (2.15-3.46)	<.0001	1.72 (1.37-2.15)	<.0001
	Medium-dose users (0.5-1.5 DDD per day)	65 (0.1)	39376	1.7	2.88 (2.21-3.75)	<.0001	1.33 (1.02-1.74)	0.033
	High-dose users (more than 1.5 DDD per day)	17 (0.1)	8000	2.1	3.67 (2.25-5.97)	<.0001	2.07 (1.27-3.38)	0.0035
Intracerebral hemorrhage	Controls	268 (0.1)	881731	0.3				
	Non-users (0 DDD per day)	205 (0.1)	712482	0.3	0.93 (0.78-1.12)	0.46	1.05 (0.87-1.26)	0.61
	Low-dose users (lower than 0.5 DDD per day)	32 (0.0)	70807	0.5	1.66 (1.12-2.48)	0.012	1.21 (0.83-1.78)	0.33
	Medium-dose users (0.5-1.5 DDD per day)	64 (0.1)	39376	1.6	5.42 (4.09-7.18)	<.0001	2.53 (1.90-3.37)	<.0001
	High-dose users (more than 1.5 DDD per day)	13 (0.1)	8000	1.6	5.24 (2.99-9.20)	<.0001	2.80 (1.59-4.93)	0.0004
Stroke UNS	Controls	746 (0.3)	881731	0.8				
	Non-users (0 DDD per day)	387 (0.2)	712482	0.5	0.63 (0.56-0.72)	<.0001	0.79 (0.70-0.90)	0.0002
	Low-dose users (lower than 0.5 DDD per day)	174 (0.1)	70807	2.5	3.19 (2.65-3.85)	<.0001	1.90 (1.60-2.27)	<.0001
	Medium-dose users (0.5-1.5 DDD per day)	108 (0.2)	39376	2.7	3.34 (2.71-4.10)	<.0001	1.59 (1.29-1.95)	<.0001
	High-dose users (more than 1.5 DDD per day)	18 (0.1)	8000	2.3	2.71 (1.70-4.34)	<.0001	1.61 (1.01-2.59)	0.047
Sepsis	Controls	482 (0.2)	881731	0.5				
	Non-users (0 DDD per day)	530 (0.3)	712482	0.7	1.36 (1.20-1.54)	<.0001	1.46 (1.28-1.65)	<.0001
	Low-dose users (lower than 0.5 DDD per day)	168 (0.1)	70807	2.4	4.39 (3.61-5.34)	<.0001	3.00 (2.48-3.62)	<.0001
	Medium-dose users (0.5-1.5 DDD per day)	246 (0.4)	39376	6.2	11.25 (9.59-13.19)	<.0001	4.89 (4.16-5.75)	<.0001
	High-dose users (more than 1.5 DDD per day)	62 (0.4)	8000	7.8	13.64 (10.42-17.84)	<.0001	6.71 (5.12-8.81)	<.0001
Pneumonia	Controls	1481 (0.7)	881731	1.7				
	Non-users (0 DDD per day)	1181 (0.6)	712482	1.7	0.97 (0.90-1.05)	0.46	1.13 (1.05-1.23)	0.0015
	Low-dose users (lower than 0.5 DDD per day)	568 (0.3)	70807	8.0	5.46 (4.90-6.09)	<.0001	3.34 (3.01-3.71)	<.0001
	Medium-dose users (0.5-1.5 DDD per day)	433 (0.6)	39376	11.0	6.74 (6.04-7.52)	<.0001	3.01 (2.69-3.36)	<.0001
	High-dose users (more than 1.5 DDD per day)	98 (0.6)	8000	12.3	7.32 (5.95-8.99)	<.0001	3.82 (3.10-4.70)	<.0001

*The cause of death can be both the primary cause of death and contributing causes of death, so the total number of deaths is therefore higher than the total deaths due to disease-specific deaths.

**Hazard ratio adjusted for comorbidities (diabetes ICD-10 code E10-E14, deep vein thrombosis I80-I82, pulmonary embolism I26, hypertension (more than 2 dispensed prescription of antihypertensive drug), stroke I64, ischemic heart disease I20-I25, heart failure I50, pneumonia J12-J18, malignant neoplasm C00-C97).

†Cerebral infarction, intracerebral hemorrhage, and stroke not specified as hemorrhage or infarction.

CI, confidence interval; GC, glucocorticoid; UNS, unspecified; ICD, International Classification of Diseases.

Mortality analysis was performed for controls and for non-users (0 DDD per day), low-dose users (more than 0 DDD per day but lower than 0.5 DDD per day), medium-dose users (0.5-1.5 DDD per day) and high-dose users (more than 1.5 DDD per day).

## Discussion

In this population-based matched cohort study of 223 211 oral GC users, we found an increased all-cause mortality compared to controls. The study illustrates that patients receiving oral GC treatment have a two-fold overall risk of dying during follow-up than matched controls, mainly due to deaths from pulmonary embolism, pneumonia, and sepsis.

Previous studies have shown that oral GC use is associated with an increased mortality rate in patients with chronic inflammatory diseases (12–14, 21). These studies showed a hazard ratio for all-cause mortality of 2.17 (95% CI 2.04 to 2.31) in patients with asthma (14), 2.48 (95% CI 1.85 to 3.31) in patients with Crohn’s disease, 2.81 (95% CI 2.26 to 3.50) in patients with ulcerative colitis (13), and 1.97 (95% CI 1.81 to 2.15) in patients with rheumatoid arthritis (21). The almost doubled risk of death is in agreement with our findings, although our results derive from a population-based cohort and cannot be directly compared to previous studies. A recent population-based cohort study showed that chronic (≥30 days) GC users had a 1.4-fold higher 5-year all-cause mortality compared with controls (16). For chronic high-dose GC users (>5 mg/day of prednisolone) the hazard ratio was 1.5 (95% CI 1.3 to 1.8; P < 0.001) and for low-dose GC users 1.3 (1.2 to 1.5; P < 0·001) (16).

Previous studies have not focused on pulmonary embolism as a specific cause of death in patients receiving GC treatment (9, 14, 16, 21). Malignant neoplasm and previous history of thromboembolic disease may increase the risk of pulmonary embolism. Therefore, it is important to emphasis that we adjusted for comorbidities such as cancer, as well as deep vein thrombosis and pulmonary embolism at baseline (before prescription of GCs) in the mortality analysis. The increased mortality rate from deaths due to pulmonary embolism in the current report is in line with the increased incidence of thromboembolism in patients with endogenous hypercortisolism (22, 23). Patients with endogenous hypercortisolism have increased levels of procoagulant factors and impaired fibrinolytic capacity, that leads to hypercoagulability with an up to ten-fold increased risk of venous thromboembolism (22, 24, 25). Data on the association between GC use and hypercoagulability are, however, sparse (25, 26). A population-based case-control study from Denmark showed that current systemic GC use was associated with an approximately two-fold increased incidence of both pulmonary embolism and deep vein thrombosis (26). Thus, our results are in line with these previous results and suggest that GC treatment at supraphysiological doses is associated with increased morbidity and mortality due to thromboembolic diseases.

GCs have immunosuppressive and anti-inflammatory effects that, consequently, increase the susceptibility to infections (27, 28). Our study showed a six-fold risk of death from sepsis and three-fold risk of death from pneumonia in high GC-dose users, and that the risk of death from sepsis is dose dependent. Patients with endogenous hypercortisolism also have an increased risk of dying from infections (29, 30). In a recent nationwide study on patients with Cushing disease, 11% of all deaths were due to infections and half of them due to pneumonia (30). Furthermore, according to a recent study from the European Register on Cushing’s syndrome (ERCUSYN), one-third of all deaths were due to infections (29). These, and our data, strongly indicate that the immunosuppressive effects of GCs may have deleterious consequences for patients with endogenous hypercortisolism as well as GC users.

Our study showed increased mortality from ischemic heart disease and heart failure, although not in dose-dependent pattern. GC use has in fact previously been associated with increased morbidity and mortality from cardiovascular disease (8, 9). GC use in patients with rheumatoid arthritis has been associated with a dose-dependent increase in cardiovascular mortality rates, with a daily threshold dose of 8 mg of Prednisolone (9). Another study showed a dose-dependent relation between current users of GC and risk of heart failure (adjusted odds ratio 2.66, 95% CI 2.46 to 2.87), and ischemic heart disease (adjusted odds ratio 1.20, 95% CI 1.11 to 1.29) (8). In our study, the hazard ratio was highest in low-dose users for deaths both from heart failure and ischemic heart disease. Due to this, and the retrospective design, a causal role between GC treatment and the increased mortality from cardiovascular diseases can however not be confirmed.

Previous studies have shown that prednisolone doses lower than 5 mg/day do not increase mortality and do not suppress the hypothalamic-pituitary-adrenal axis (21, 31). Similarly, GC treatment for less than 2-3 weeks does not seem to suppress the hypothalamic-pituitary-adrenal axis (32–34). Patients receiving prednisolone equivalent doses of <5 mg/day for <21 days were therefore not included in our study. On the contrary, higher doses and/or longer treatment duration frequently causes transient GC-induced cortisol deficiency, also called GC-induced adrenal insufficiency (6). In such cases, GC cessation can be hazardous and lead to acute adrenal crisis (35). A recent study including 70,638 oral GC users showed increased mortality during the first 2 months after cessation of oral GC treatment and then decreased mortality over time after the first 3 months of cessation (15). This may have been caused by adrenal crisis due to undiagnosed GC-induced adrenal insufficiency. An increased mortality in GC users due to sepsis and pneumonia could be related to adrenal crisis. However, this is only speculative since our data does not contain information on whether the GC treatment was tapered slowly or not. More studies are needed to investigate if GC-induced adrenal insufficiency is underdiagnosed in GC users and whether it is associated with premature and avoidable death.

The main strength of our study is the access to large healthcare databases with information about dispensed prescriptions at all Swedish pharmacies, causes of death, and comorbidities. The Swedish Prescribed Drug Register has information on all dispensed prescriptions in Sweden offering the opportunity to evaluate mortality in oral GC users, both adults and children, in a large population-based cohort, in contrast to previous studies with focus on mortality in GC users with specific diseases (rheumatoid arthritis, inflammatory bowel disease, asthma, and chronic inflammatory disease) (12–14, 21). However, the true causal relationship between oral GC use and mortality is challenging to uncover due to a large number of confounders, including the underlying disease itself and its severity (9, 10). In fact, high-dose GC users are more likely to have more severe underlying diseases than low-dose users, that consequently may explain the increased mortality rate. Previous studies that have investigated mortality in GC treated patients with one specific disease have the same limitations and a true causal relationship between GC use and mortality can therefore not be proven (10, 12, 14). Further research on this topic is therefore needed.

This large matched cohort study showed that oral GC users have a high all-cause mortality compared to the background population, mainly due to deaths from sepsis, pulmonary embolism, and heart failure.

## Data Availability Statement

The raw data supporting the conclusions of this article will be made available by the authors, without undue reservation.

## Ethics Statement

The studies involving human participants were reviewed and approved by The Regional Research Ethics Committee in Gothenburg, Sweden (reference number 773-14; approved 9 March 2015) and by the National Board of Health and Welfare, Sweden. Written informed consent for participation was not required for this study in accordance with the national legislation and the institutional requirements.

## Author Contributions

ME, OR, GJ, DO, and PT designed the study. OR supervised the study. PE and MM had full access to data in the study and performed the statistical analysis. GJ and ME obtained funding. ME and OR drafted the manuscript and all authors revised it. All authors approved the final manuscript. OR and ME are guarantors. All authors contributed to the article and approved the submitted version.

## Funding

ME was supported by a grant from the Gothenburg Medical Society (grant number 17/691951). The study was conducted with research grants from The Healthcare Committee, Region Västra Götaland (grant numbers 15/573411 and 17/751841).

## Conflict of Interest

DO has received consultant fees from Pfizer, Sandoz, Ipsen and NovoNordisk. Unrestricted project grants from Sandoz and Pfizer. Since the 30th of Aug 2021 been employed by AstraZeneca. GJ has served as consultant for Shire and Astra Zeneca, and has received lecture fees from Eli Lilly, Ipsen, Novartis, Novo Nordisk, Merck Serono, Otsuka, and Pfizer.

The remaining authors declare that the research was conducted in the absence of any commercial or financial relationships that could be construed as a potential conflict of interest.

## Publisher’s Note

All claims expressed in this article are solely those of the authors and do not necessarily represent those of their affiliated organizations, or those of the publisher, the editors and the reviewers. Any product that may be evaluated in this article, or claim that may be made by its manufacturer, is not guaranteed or endorsed by the publisher.
